# More Than 100 Persistent Symptoms of SARS-CoV-2 (Long COVID): A Scoping Review

**DOI:** 10.3389/fmed.2021.750378

**Published:** 2021-11-01

**Authors:** Lawrence D. Hayes, Joanne Ingram, Nicholas F. Sculthorpe

**Affiliations:** ^1^School of Health and Life Sciences, Institute of Clinical Exercise and Health Science, University of the West of Scotland, Hamilton, United Kingdom; ^2^School of Education and Social Sciences, University of the West of Scotland, Paisley, United Kingdom

**Keywords:** coronavirus–COVID-19, COVID-19, long COVID, SARS-CoV-2, persistent, symptoms, post acute covid syndrome (PACS)

## Abstract

**Background:** Persistent coronavirus disease 2019 (COVID-19) symptoms are increasingly well-reported in cohort studies and case series. Given the spread of the pandemic, number of individuals suffering from persistent symptoms, termed ‘long COVID', are significant. However, type and prevalence of symptoms are not well reported using systematic literature reviews.

**Objectives:** In this scoping review of the literature, we aggregated type and prevalence of symptoms in people with long COVID.

**Eligibility Criteria:** Original investigations concerning the name and prevalence of symptoms were considered in participants ≥4-weeks post-infection.

**Sources of Evidence:** Four electronic databases [Medline, Web of Science, Scopus, and the Cochrane Central Register of Controlled Trials (CENTRAL)] were searched.

**Methods:** A scoping review was conducted using the Arksey and O'Malley framework. Review selection and characterisation was performed by three independent reviewers using pretested forms.

**Results:** Authors reviewed 2,711 titles and abstracts for inclusion with 152 selected for full-text review. 102 articles were subsequently removed as this did not meet inclusion criteria. Thus, fifty studies were analysed, 34 of which were described as cohort studies or prospective cohort studies, 14 were described as cross-sectional studies, one was described as a case control study, and one was described as a retrospective observational study. In total, >100 symptoms were identified and there was considerable heterogeneity in symptom prevalence and setting of study. Ten studies reported cardiovascular symptoms, four examined pulmonary symptoms, 25 reported respiratory symptoms, 24 reported pain-related symptoms, 21 reported fatigue, 16 reported general infection symptoms, 10 reported symptoms of psychological disorders, nine reported cognitive impairment, 31 reported a sensory impairment, seven reported a dermatological complaint, 11 reported a functional impairment, and 18 reported a symptom which did not fit into any of the above categories.

**Conclusion:** Most studies report symptoms analogous to those apparent in acute COVID-19 infection (i.e., sensory impairment and respiratory symptoms). Yet, our data suggest a larger spectrum of symptoms, evidenced by >100 reported symptoms. Symptom prevalence varied significantly and was not explained by data collection approaches, study design or other methodological approaches, and may be related to unknown cohort-specific factors.

## Introduction

### Rationale

An unprecedented surge in research following the onset of the severe acute respiratory syndrome coronavirus (SARS-CoV)-2 [also termed *Coronavirus-19 (COVID-19)*] pandemic means that, despite being a relatively new condition, much is now known about acute COVID-19 presentation and management (1–9). However, as the pandemic developed, it became clear that a significant proportion of patients experienced symptoms which persisted beyond the initial viral infection. Named initially by patients themselves (10), the term long COVID has become the most commonly used phrase to describe the condition and broadly describes individuals who have recovered from acute COVID-19, but experience symptoms which are persistent or very slow to resolve (11). These individuals manage with severe and debilitating symptoms, which are often cyclical in nature with periods of remission, followed by periods of extreme symptom exacerbation (12). Moreover, because long COVID symptoms develop after the viral infection, there have been several calls to redefine recovery from COVID-19 infection as requiring more than the absence of active infection (13). A further complication is that not only are long COVID symptoms disparate from acute COVID-19 symptoms, their severity is unrelated to initial acute infection severity (14).

Long COVID symptoms are not well described, partly because this requires longitudinal tracking of individuals, and the emergence of such evidence will naturally be delayed compared to those of acute symptoms. Nevertheless, some relatively common symptoms have emerged, with effects of long COVID reported to include cardiovascular (15), pulmonary (16), and respiratory symptoms (17, 18), pain of several anatomical locations (17, 19–23), fatigue (24–26), general infection symptoms [e.g., nausea (19), diarrhoea (27), fever (28), etc.], psychological disorders (29), cognitive impairment (30), sensory impairment (31), dermatological complaints (32), and functional impairment (33). Indeed, one of the remarkable aspects of the condition is the wide variety of symptoms associated with it. Furthermore, the prevalence with which different physiological systems are involved appears to vary considerably. For example, prevalence of fatigue in people with long COVID ranges between 53% in Italy (24) and 98% in the UK (34). This divergence may be in part due to study design. For example, if an investigation is conducted in a smell and taste clinic, soon after acute COVID-19 recovery, it is likely a large proportion of participants will present with dysnosmia or dysgeusia [e.g., (35); 100% of participants]. Conversely, if an investigation includes all those recovered from acute COVID-19, months after acute COVID-19 recovery, prevalence of sensory impairment will be significantly less [e.g., (17); 11% of participants]. However, this has not been extensively examined in systematic reviews of the literature to date and therefore warrants further investigation. The two systematic reviews that exist to our knowledge (36, 37) report considerable divergence in results despite similar objectives. Indeed, Iqbal et al. (36) identify multiple flaws in data capture and interpretation, and thus urge caution in application of the meta-analytical findings.

A comprehensive review of long COVID symptoms is important for clinicians to ensure they can support individuals with appropriate care and prescription. As such, it seemed pragmatic to conduct a scoping review in this area to map existing literature in terms of the volume, nature, and characteristics of the primary research (38). We used a scoping review rather than systematic review and meta-analysis because 1) our aim was to characterise symptoms of long COVID as reported in the available literature, rather than pose a specific and focused research question (39), and 2) the wide variations in study designs, inclusion criteria, and sampling meant effective pooling of data was unlikely to be feasible (40). A comprehensive review of long COVID symptoms is an essential tool to guide clinical decision making. However, a standard systematic review requires a strong understanding of the area to which specific research questions can be addressed. Given reports of broad heterogeneity in symptoms, severity, and prevalence, and that clear diagnostic criteria for long COVID are not yet established, our understanding of the development and symptoms of long COVID is not sufficient to develop such a question. As such, a traditional systematic review and meta-analysis would have been premature. Consequently, we elected to undertake a scoping review as the current state of the literature was relatively unknown in terms of methodologies and data reporting. This approach retains the systematic approach to literature searching but aims to map out a new and rapidly developing area where a consensus of findings may be unlikely (39). Using the framework of Arksey and O'Malley, a scoping review aims to use a broad set of search terms and include a wide range of study designs and methods [in contrast to a systematic review (38)]. This approach, however, has the benefit of clarifying key concepts, surveying current data collection approaches, and identifying critical knowledge gaps.

### Objectives

We aimed to provide an overview of existing literature concerning long COVID symptoms. Our three specific objectives of this scoping review were to 1) conduct a systematic search of the published literature concerning long COVID symptoms and their prevalence, 2) map characteristics and methodologies used, and 3) provide recommendations for the advancement of the investigative area.

## Methods

### Protocol and Registration

The review was conducted and reported according to the Preferred Reporting Items for Systematic Reviews and Meta-Analyses extension for scoping reviews (PRISMA-ScR) guidelines (41) and the five-stage framework outlined in Arksey and O'Malley (38). A review protocol was not published.

### Eligibility Criteria

Studies that met the following criteria were included: (1) involvement of human participants; (2) not a review; (3) an investigation which considered participants ≥4-weeks after acute COVID-19 infection (COVID-19 rapid guideline: managing the long-term effects of COVID-19; NICE); (4) employed a study design which was not a case study or case series; (5) published in English; (6) including outcome measures related to (i) symptoms, and (ii) symptom prevalence.

### Search Strategy

The search strategy consisted of a combination of free-text and MeSH terms relating to persistent symptoms following COVID infection which were developed through examination of published original literature and review articles. Example search terms for PubMed included: (COVID or COVID-19 OR Sars-Cov-2) AND (long COVID OR persistent symptoms OR post-acute OR post-viral).

### Information Sources

Four electronic databases [Medline, Web of Science, Scopus, and the Cochrane Central Register of Controlled Trials (CENTRAL)] were searched to identify original research articles published from the earliest available date up until 5th February 2021. Additional records were identified through reading included studies.

### Study Selection and Data Items

Data were extracted by three reviewers (LH, JI, and NS) independently and compared in an unblinded and standardised manner. Once each database search was completed and manuscripts sourced, studies were downloaded into a single reference list with duplicates removed. Titles and abstracts were then screened for eligibility and full texts were only retrieved for studies with symptom prevalence incorporated. Full texts were then assessed using the complete eligibility criteria with all authors confirming inclusion and exclusion. Following this assessment, the same reviewers read the studies and assessed the following: design method, participant characteristics, setting, study duration, and symptoms. Descriptions were extracted with as much detail provided by the authors. Any uncertainty by reviewers was discussed in consensus meetings and resolved by agreement. Data extracted from each study included sample size, group descriptions, study design, and outcome data. The primary outcome variables were defined as symptom type and symptom prevalence.

## Results

### Study Selection

**Figure 1:**
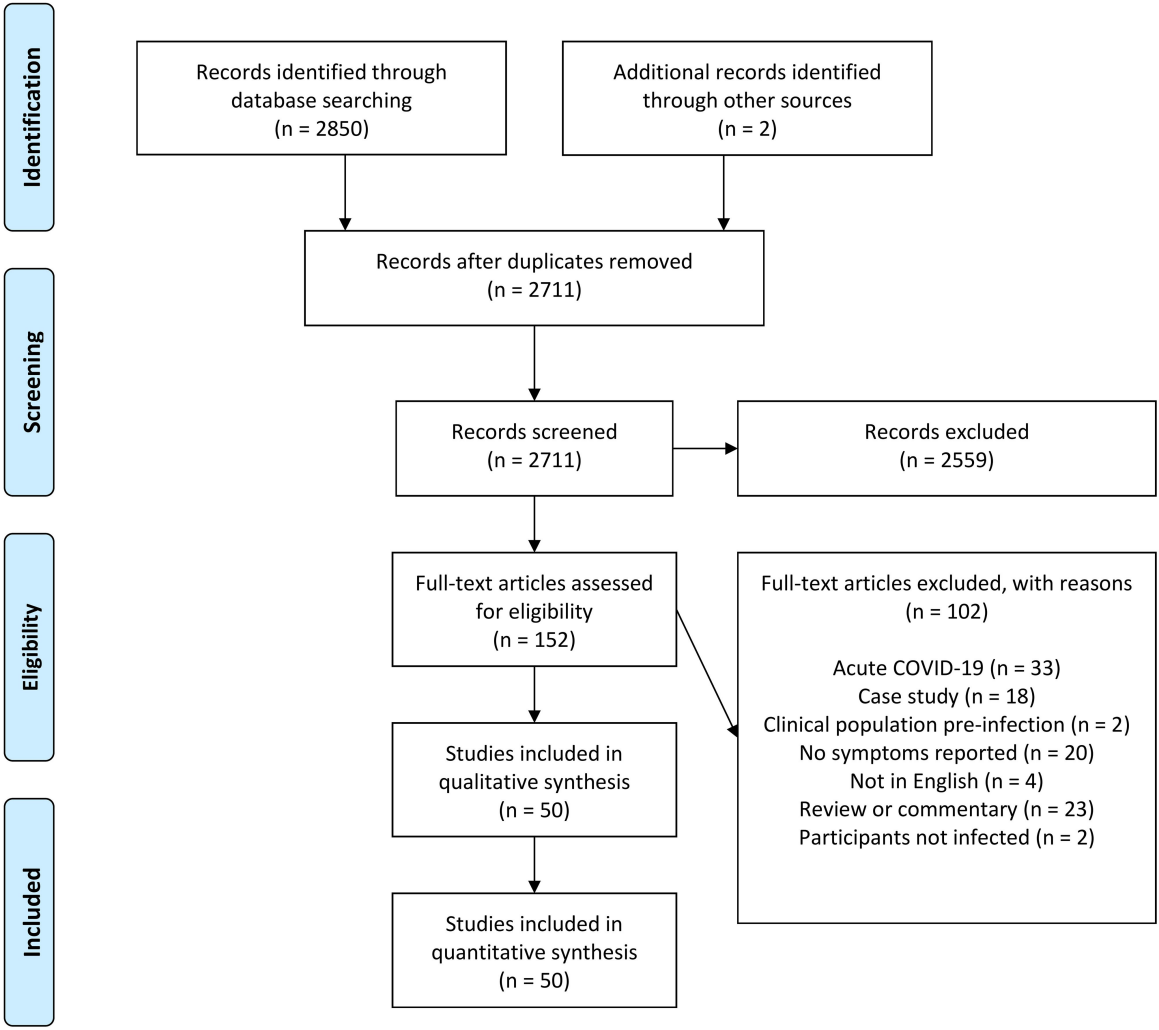
Schematic flow diagram describing exclusions of potential studies and final number of studies.

After the initial database search, 2,852 records were identified (Figure 1). Once duplicates were removed, 2,711 titles and abstracts remained, and titles and abstracts were screened for inclusion resulting in 152 full-text articles being sourced and screened. Of these, 102 were excluded and 50 remained.

### Study Characteristics

Of the 50 studies included, 34 were described as cohort studies or prospective cohort studies, 14 were described as cross-sectional studies, one was described as a case control study, and one was described as a retrospective observational study (Table 1). Where a study had multiple symptoms described, they were extracted separately and grouped by symptom, rather than study (Table 2).

**Table 1:** General study information of studies concerning long COVID symptoms.

**References**	**Study** **design**	**Sample** **size**	**Study** **registration** **(Y/N)**	**Gender** **split**	**Country** **of study**	**Hospitalised** **(Y/N)/ICU or** **general ward/length** **of stay**	**Time from** **acute infection** **(days)**	**Participant** **age (yrs)**
Bellan et al. (29)	Cohort	238	N	Unclear	Italy	Y/unclear/unclear	122	61 (50–70)
Boscolo-Rizzi et al. (27)	Cross sectional	202 (187 post)	N	45% male	Italy	Unclear/unclear/unclear	28	56 (20–89)
Cai et al. (42)	Cohort	126	N	48% male	China	Y/unclear/25 d	39 ± 7	46 ± 14
Carfi et al. (24)	Cohort	143	N	63% male	Italy	Y/both (14% ICU)/14 ± 10 d	60 ± 13	56 ± 15
Caronna et al. (43)	Cohort	130 (97 with headache)	N	49% male	Spain	Both (80% hospitalised)/unclear/unclear	42	54 ± 16
Carvalho-Schneider et al. (44)	Cohort	150	N	44% male	France	Both/unclear/unclear	30 60	49 ± 15 49 ± 15
Chiesa-Estomba et al. (45)	Cohort	751		36% male	France	Both/unclear/unclear	47	41 ± 13
Curci et al. (46)	Cohort	41	N	61% male	Italy	Y/ICU/18 ± 9 d	31 ± 9	72 ±11
Dennis et al. (28)	Cohort	201	Y	30% male	UK	Both (18% hospitalised)/unclear/unclear	91	44 ± 11
Fjaeldstad (47)	Cohort	109 but only 42 confirmed C-19	N	24% male	Denmark	N	> 30	37 (34–41)
Frija-Masson et al. (48)	Cohort	50	N	66% male	France	Y/unclear/unclear	30	54 (42–62)
Galván-Tejada et al. (49)	Case Control	141 C-19 positive and 78 Control	N	49% male	Mexico	Unclear/unclear/unclear	Possibly 60 but unclear	39
Garrigues et al. (32) (ICU) (ward)	Cohort	96 24	N N	58% male 79% male	France France	Y/general ward/7 ± 5 d Y/ICU/27 ± 22 d	>100 >100	Unclear Unclear
Goërtz et al. (19) (Hospitalised) (confirmed C-19)	Cohort	112 354	N N	30% male 9% male	Netherlands Netherlands	Y/unclear/unclear N	79 ± 17 79 ± 17	53 (46–60) 47 (34–54)
Hall et al. (50)	Cohort	200	N	62% male	UK	Y/ICU/9 d	28–42	55 ± 15
Huang et al. (17)	Cohort	1,733	N	52% male	China	Y/both/14 (10–19) d	186 [175–199]	57 (47–65)
Huang et al. (51)	Observational	26 C-19 positive and 20 control	N	38% male	China	Y/unclear/unclear	47 [36–58]	38 (32–45)
Iannuzzi et al. (52)	Cohort	34	N	47% male	Italy	Y/general ward/unclear	~61	48 ± 13
Jacobs et al. (20)	Cohort	183	N	62% male	USA	Y/unclear/≥3 d	35 ± 5	57 (48–68)
Janiri et al. (53)	Cross sectional	61	N	Unclear	Italy	Y/both/~17 d	41 ± 19	~66 ± 6
Kamal et al. (54)	Cross sectional	287	N	36% male	Egypt	Both/unclear/unclear	Unclear	32 ± 9
Kandemirli et al. (35)	Cohort	23	N	39% male	Unclear	Unclear/unclear/unclear	>30	29 (21–41)
Konstantinidis et al. (55)	Cross sectional	79	N	53% male	Greece	Unclear/unclear/unclear	28	31 ± 5
Machado et al. (16)	Cross sectional	1,939	N	15% male	Netherlands and Belgium	Unclear/general ward/unclear	79 ± 17	46 ± 11
Mandal et al. (56)	Cross sectional	384	N	62% male	UK	Y/both/7 (4–11) d	54 [47–59]	60 ± 16
Mannan et al. (18)	Multi-centre cross sectional	1,021	N	75% male	Bangladesh	Unclear (some asymptomatic)/unclear/unclear	>28	<9->60
Mazza et al. (57)	Prospective cohort study	402	N	66% male	Italy	Y/ICU/15 ± 10 d	31 ± 16	58 ± 13
Mendez et al. (58)	Cross sectional	179	N	59% male	Spain	Y/both/7 (9–18) d	61 ± 30	57 [49–67]
Meys et al. (21)	Cross sectional	210	N	12% male	Netherlands and Belgium	Unclear/unclear/unclear	79 ± 17	45 ± 11
Moreno-Perez et al. (59)	Prospective cohort study	277	N	53% male	Spain	Y/both/9 (6–12) d	Range 70–98	56 [42–68]
Munro et al. (60)	Prospective cohort study	121	N	Unclear	UK	Both/unclear/unclear	~56	64 (range 44–82)
Niklassen et al. (61)	Multi-centre prospective cohort	111	N	53% male	Italy	Unclear/unclear/unclear	63 ± 46	Grouped 18–39, 40–69. 70+
Ortelli et al. (62)	Cross sectional	12 C-19 positive and 12 control	N	83% male	Italy	Y/general ward/unclear	63 - 91	67 ± 10
Petersen et al. (63)	Prospective cohort study	180	N	56% male	Faroe Islands	Both/unclear/2 (range 0–11) d	125 (range 45–153)	40 ± 19
Poncet-Megemont et al. (64)	Cohort	139	N	37% male	France	Both/unclear/unclear	30–35	49 ± 15
Printza et al. (65)	Cohort	90	N	59% male	Greece	Y/unclear/unclear	61 [IQR 7]	56 ± 17
Raman et al. (66)	Cohort	58 C-19 positive and 30 control	N	59% male	UK	Y/both (36% ICU)/9 (5–17) d	70 [63–77]	55 ± 13
Shah et al. (67)	Prospective cohort study	60	N	68% male	Canada	Y/unclear/10 (6–16) d	82 (range 56–84)	67 [54–74]
Sonnweber et al. (68)	Prospective cohort study	145	N	55% male	Austria	Both/both (22% ICU)/unclear	63 ± 23 103 ± 21	57 ± 14
Stavem et al. (31)	Cross sectional	451	N	44% male	Norway	N	117 (range 41–193)	50 ± 15
Taboada et al. (69)	Cohort	183	N	unclear	Spain	Y/both (18% ICU)/unclear	183	unclear
Taboada et al. (25)	Prospective cohort study	91	N	65% male	Spain	Y/ICU/35 ± 21 d	183	66 ± 10
Tomasoni et al. (22)	Cross sectional	105	N	73% male	Italy	Y/both/8 (6–11) d	46 [43–48]	55 [43–65]
Townsend et al. (26)	Prospective cohort study	128	N	46% male	Ireland	Both (56% hospitalised)/unclear/unclear	72 [62–87]	50 ± 15
Trickmann et al. (23)	Prospective cohort study	246	N	Unclear	Germany	Both/both (≤ 1% ICU)/unclear	68 ± 16	48 ± 15
van den Borst et al. (33)	Prospective cohort study	124 (including 17 without a positive test)	N	60% male	The Netherlands	Both (86% hospitalised)/unclear/8 (5–14) d	91 ± 14	59 ± 14
Woo et al. (30)	Cross sectional	18 C-19 positive and 10 control	N	42% male	Germany	Both/general ward/unclear	85 (range 20–105)	42 (range 17–71)
Xiong et al. (15)	Prospective cohort study	538 C-19 positive and 184 control	N	46% male	China	Y/unclear/unclear	97 [95–102]	52 [41–62]
Yan et al. (70)	Cross sectional	46	N	unclear	USA	Unclear/unclear/unclear	unclear	unclear
Zhao et al. (71)	Cohort	55	N	58% male	China	Y/unclear/unclear	64–93	48 ± 15

*C-19, COVID-19; d, day; Y, yes; N, no; ICU, intensive care unit. Data are presented as mean ± standard deviation, median [interquartile range], or mean (range)*.

**Table 2:** Summary of long COVID symptoms, grouped by category, with prevalence reported in each study and studies listed in order of prevalence for each symptom.

**Symptoms**	**Incidence or** **proportion (reference)**	**Time from acute** **infection (days)**
**Cardiovascular**		
Myocardial oedema	54% of 26 (51)	47 [36–58]
Palpitations	55.4% of 354 [(19) – Non-hospitalised]	79 ± 17
	39.3% of 112 [(19) – Hospitalised]	79 ± 17
	10.9% of 130 (44)	60
	6.5% of 150 (44)	30
	9% of 1,733 (17)	186 [175–199]
“Deterioration of cardiac causes”/	32% of 201 (28)	91
Cardiac impairment	4% of 200 (50)	28–42
Positive CMR findings	58% of 26 (51)	47 [36–58]
Stroke	3% of 287 (54)	Unclear
Myocarditis	1% of 287 (54)	Unclear
Arrythmia	<1% of 287 (54)	Unclear
Elevated heart rate	57.7% of 354 [(19) – Non-hospitalised]	79 ± 17
	51.8% of 112 [(19) – Hospitalised]	79 ± 17
	4.8% of 538 (15)	97 [95 – 102]
Pericardial effusion	6% of 145 (68)	63 ± 23
	1% of 145 (68)	103 ± 21
Diastolic dysfunction	60% of 145 (68)	63 ± 23
	55% of 145 (68)	103 ± 21
Newly diagnosed hypertension	1.3% of 538 (15)	97 [95–102]
**Pulmonary**		
Pulmonary embolus	2% of 200 (50)	28–42
Lung infarcts	1% of 200 (50)	28–42
Pulmonary fibrosis	5% of 287 (54)	Unclear
Chest imaging abnormalities	88% of 60 (67)	82 (range 56–84)
	77% of 145 (68)	63 ± 23
	63% of 145 (68)	103 ± 21
Signs of pulmonary hypertension	10% of 145 (68)	63 ± 23
	10% of 145 (68)	103 ± 21
Impaired lung function	42% of 145 (68)	63 ± 23
	36% of 145 (68)	103 ± 21
**Respiratory**		
Dyspnoea/breathlessness/shortness of breath/breathing problems	87.1% of 201 (28)	91
	87% of 354 [(19) – Non-hospitalised]	79 ± 17
	80.3% of 112 [(19) – Hospitalised]	79 ± 17
	71% of 183 (20)	35 ± 5
	64% of 58 (66)	70 [63–77]
	64% of 210 (21)	79 ± 17
	57.1% of 91 (69) * on exertion	183
	55% of 384 (56)	54 [47–59]
	50.3% of 96 [(32) – ICU]	>100
	42.8% of 143 (24)	60 ± 13
	39.6% of 24 [(32) – Ward]	>100
	39% of 187 (27)	28
	36% of 145 (68)	100
	34% of 227 (59)	Range 70–98
	32% of 246 (23)	68 ± 16
	29% of 1,021 (18)	>28
	28% of 287 (54)	Unclear
	28% of 1,939 (16)	79 ± 17
	26.4% of 91 (25) *on slight exertion	183
	26% of 1,733 (17)	186 [175–199]
	20% of 60 (67)	82 (range 56–84)
	16% of 451 (31)	117 (range 41–193)
	14.5% of 55 (exertional) (71) *exertional	Range 64–93
	10.7% of 130 (44)	30
	7.7% of 130 (44)	60
	6.7 or 26% of 105 (22)^***^	46 [43–48]
	5.5% of 238 (29)	122
	NR of 180 (63)	125 (range 45–153)
Cough	79.5% of 112 [(19) – Hospitalised]	79 ± 17
	73.6% of 201 (28)	91
	68.1% of 354 [(19) – Non-hospitalised]	79 ± 17
	63% of 1,021 (18)	>28
	61% of 183 (20)	35 ± 5
	55% of 384 (56)	54 [47–59]
	39.7% of 187 (27)	28
	25% of 96 [(32) – ICU]	>100
	21% of 227 (59)	Range 70–98
	20% of 60 (67)	82 (range 56–84)
	17% of 145 (68)	100
	15.8% of 143 (24)	60 ± 13
	14.6% of 24 [(32) – ward]	>100
	14.4% of 91 (25)	183
	14% of 246 (23)	68 ± 16
	7.1% of 538 (15)	97 [95–102]
	1.8% of 55 (71)	Range 64–93
	NR of 180 (63)	125 (range 45–153)
	NR of 1,939 (16)	79 ± 17
	NR of 451 (31)	117 (range 41–193)
Runny nose	49.0 of 354% [(19) – Non-hospitalised]	79 ± 17
	33.9% of 112 [(19) – Hospitalised]	79 ± 17
	33.8% of 201 (28)	91
	21% of 1,021 (18)	>28
	12.8% of 143 (24)	60 ± 13
	<1% of 246 (23)	68 ± 16
	NR of 451 (31)	117 (range 41–193)
	NR of 180 (63)	125 (range 45–153)
Sore throat	71.1% of 201 (28)	91
	54.5% of 354 [(19) – Non-hospitalised]	79 ± 17
	43.8% of 112 [(19) – Hospitalised]	79 ± 17
	27% of 1,021 (18)	>28
	13.6% of 187 (27)	28
	10% of 143 (24)	60 ± 13
	4% of 17 (17)	186 [175–199]
	3.2% of 538 (15)	97 [95–102]
	<1% of 246 (23)	68 ± 16
	0% of 238 (29)	122
	NR of 451 (31)	117 (range 41–193)
	NR of 180 (63)	125 (range 45–153)
Dry mouth	NR of 17 (16)	79 ± 17
Dysphagia	NR of 17 (16)	79 ± 17
Low FVC	27% of 145 (68)	63 ± 23
	22% of 145 (68)	103 ± 21
	13% of 58 (66)	70 [63–77]
	10.9% of 55 (71)	Range 64–93
Impaired spirometry	13% of 227 (59) *restriction	Range 70–98
	4% of 227 (59) *global	Range 70–98
	2% of 227 (59) *obstruction	Range 70–98
Phlegm	37.5% of 112 [(19) – Hospitalised]	79 ± 17
	35% of 183 (20)	35 ± 5
	31.9% of 354 [(19) – Non-hospitalised]	79 ± 17
	7.9% of 143 (24)	60 ± 13
	3% of 538 (15)	97 [95–102]
Blocked nose	22.9% of 187 (27)	28
Sino-nasal pain	9.7% of 187 (27)	28
Wheezing	48.3% of 201 (28)	91
	NR of 451 (31)	117 (range 41–193)
Pain/ burning in lungs	47.3% of 112 [(19) – Hospitalised]	79 ± 17
Sneezing	35.7% of 354 [(19) – Non-hospitalised]	79 ± 17
	24.1% of 113 [(19) – Hospitalised]	79 ± 17
Polypnoea	21.4% of 538 (15) *post-activity	97 [95–102]
	4.7% of 538 (15) *nonmotor	97 [95–102]
Chest Distress	14.1% of 538 (15)	97 [95–102]
**Pain**		
Chest pain	73.1% of 201 (28)	91
	62.6% of 354 [(19) – Non-hospitalised]	79 ± 17
	47.3% of 112 [(19) – Hospitalised]	79 ± 17
	29% of 287 (54)	Unclear
	21.7% of 143 (24)	60 ± 13
	20% of 1,021 (18)	>28
	18.0% of 150 (44)	30
	13.1% of 130 (44)	60
	12.3% of 538 (15)	97 [95–102]
	11.5% of 24 [(32) – ward]	>100
	9.7% of 187 (27)	28
	8.8% of 91 (25)	183
	8.3% of 96 [(32)–ICU]	>100
	0.4% of 238 (29)	122
	NR of 180 (63) *chest tightness	125 (range 45–153)
Headache	83% of 201 (28)	91
	79.1% of 354 [(19) – Non-hospitalised]	79 ± 17
	71.4% of 112 [(19) – Hospitalised]	79 ± 17
	37.8% of 130 (43)	42
	33% of 183 (20)	35 ± 5
	29% of 287 (54)	Unclear
	23.7% of 187 (27)	28
	18.8% of 55 (71)	Range 64–93
	18% of 227 (59)	Range 70–98
	8.7% of 143 (24)	60 ± 13
	3.6% of 139 (64)	Range 30–35
	2% of 17 (17)	186 [175–199]
	0% of 238 (29)	122
	<1% of 246 (23) *cephalgia	68 ± 16
	NR of 17 (16)	79 ± 17
	NR of 451 (31)	117 (range 41–193)
	NR of 180 (63)	125 (range 45–153)
Joint pain/arthralgia	78.1% of 201 (28)	91
	47.3% of 112 [(19) – Hospitalised]	79 ± 17
	43.8% of 354 [(19) – Non-hospitalised]	79 ± 17
	31% of 287 (54)	Unclear
	30% of 183 (20)	35 ± 5
	28.6% of 91 (25)	183
	27% of 143 (24)	60 ± 13
	16.3% of 130 (44)	60
	9.8% of 150 (44)	30
	9% of 1,733 (17)	186 [175–199]
	7.6% of 538 (15)	97 [95–102]
	NR of 180 (63)	125 (range 45–153)
Muscle pain/myalgia	53.6% of 354 [(19) – Non-hospitalised]	79 ± 17
	43% of 183 (20)	35 ± 5
	37.4% of 91 (25)	183
	33% of 112 [(19) – Hospitalised]	79 ± 17
	6 and 10% of 143 (24)	60 ± 13
	4.5% of 538 (15)	97 [95–102]
	2% of 1,733 (17)	186 [175–199]
	NR of 451 (31)	117 (range 41–193)
Migraine	3% of 287 (54)	Unclear
Pain or discomfort	70% of 210 (21)	79 ± 17
	53.7% of 201 (28)	91
	48% of 91 (25)	183
	24% of 145 (68)	100
	10.5% of 105 (22) *burning pain	46 [43–48]
	8.7% of 187 (27)	28
	2% of 1,733 (17)	186 [175–199]
	NR of 1,939 (16)	79 ± 17
Myalgias-arthralgias	71% of 354 [(19) – Non-hospitalised]	79 ± 17
	68% of 201 (28)	91
	53.6% of 112 [(19) – Hospitalised]	79 ± 17
	20% of 187 (27)	28
	20% of 227 (59)	Range 70–98
	5.9% of 238 (29) *arthralgia	122
	5.9 % of 238 (29) * myalgia	122
Ear pain	21.4% of 354 [(19) – Non-hospitalised]	79 ± 17
	10.7% of 112 [(19) – Hospitalised]	79 ± 17
	NR of 451 (31)	117 (range 41–193)
Abdominal pain	NR of 451 (31)	117 (range 41–193)
Thoracic pain	6% of 246 (23)	68 ± 16
Limb pain	1% of 246 (23)	68 ± 16
Limb odema	2.6% of 538 (15)	97 [95–102]
**Fatigue**		
Muscle weakness	63% of 1,733 (17)	186 [175–199]
	NR of 1,939 (16)	79 ± 17
	37.4% of 91 (25)	183
	31.4% of 105 (22)	46 [43–48]
Fatigue/tiredness	93.9% of 354 [(19) – Non-hospitalised]	79 ± 17
	92.9% of 112 [(19) – Hospitalised]	79 ± 17
	90% of 201 (28)	91
	83% of 183 (20)	35 ± 5
	69% of 124 (33)	91 ± 14
	67% of 384 (56)	54 [47–59]
	58.3% of 96 [(32) – ICU]	>100
	55% of 58 (66)	70 [63–77]
	54.2% of 24 [(32) – Ward]	>100
	53% of 143 (24)	60 ± 13
	52.3% of 128 (26)	72 [62–87]
	35% of 227 (59)	Range 70–98
	28.3% of 538 (15)	97 [95–102]
	16.7% of 18 (30)	85 (range 20–105)
	16.4% of 55 (71)	Range 64–93
	14% of 1,021 (18)	>28
	13.9% of 187 (27)	28
	1% of 246 (23)	68 ± 16
	NR of 1,939 (16)	79 ± 17
	NR of 180 (63)	125 (range 45–153)
Lack of energy	5.6% of 18 (30)	85 (range 20–105)
**General infection**		
Nausea	45.5% of 112 [(19) – Hospitalised]	79 ± 17
	35.9% of 354 [(19) – Non-hospitalised]	79 ± 17
	2.6% of 187 (27)	28
	NR of 1,939 (16)	79 ± 17
	NR of 180 (63)	125 (range 45–153)
Diarrhoea	59.2% of 201 (28)	91
	50.0% of 150 (44)	30
	43.8% of 112 [(19) – Hospitalised]	79 ± 17
	43.5% of 354 [(19) – Non-hospitalised]	79 ± 17
	36% of 183 (20)	35 ± 5
	33.3% of 130 (44)	60
	22% of 1,021 (18)	>28
	11.9% of 187 (27)	28
	11% of 227 (59)	Range 70–98
	9% of 145 (68) *diarrhoea or vomiting	100
	5% of 1,733 (17) *diarrhoea or vomiting	186 [175–199]
	2.8% of 143 (24)	60 ± 13
	1.3% of 238 (29)	122
	NR of 451 (31)	117 (range 41–193)
	NR of 180 (63)	125 (range 45–153)
Upset stomach	30.9% of 55 (71) *gastrointestinal symptoms	Range 64–93
	1% of 105 (22)	46 [43–48]
Fever	83.9% of 112 [(19) – Hospitalised]	79 ± 17
	81% of 1,021 (18)	>28
	75.1% of 201 (28)	91
	51.6% of 354 [(19) – Non-hospitalised]	79 ± 17
	20% of 183 (20)	35 ± 5
	11% of 287 (54)	Unclear
	4.8% of 187 (27)	28
	3.6% of 150 (44)	30
	1% of 451 (31)	117 (range 41–193)
	<1% of 20 (17)	186 [175–199]
	<1% of 246 (23)	68 ± 16
	0% of 238 (29)	122
	0% of 130 (44)	60
	NR of 1,939 (16)	79 ± 17
Ulcer	6% of 183 (20)	35 ± 5
Vomiting	13% of 20 (18)	>28
	11.9% of 354 [(19) – Non-hospitalised]	79 ± 17
	9% of 112 [(19) – Hospitalised]	79 ± 17
	0% of 187 (27)	28
	NR of 451 (31)	117 (range 41–193)
Chills	4.6% of 538 (15)	97 [95–102]
	NR of 451 (31)	117 (range 41–193)
	NR of 180 (63)	125 (range 45–153)
**Psychological**		
PTSD	42.9% of 238 (29)	122
	31% of 126 (42)	39 ± 7
	25% of 179 (58)	61 ± 30
	15% of 402 (57) *IES-R	31 ± 16
	7% of 124 (33)	91 ± 14
Distress	30% of 61 (53)	41 ± 19
	12% of 124 (33) *stress reaction to traumatic event	91 ± 14
Anxiety	42% of 402 (57) *state anxiety	31 ± 16
	36% of 402 (57) *trait anxiety	31 ± 16
	38% of 287 (54)	Unclear
	30% of 179 (58)	61 ± 30
	29% of 105 (22)	46 [43–48]
	22.2% of 126 (42)	39 ± 7
	14% of 58 (66)	70 [63–77]
	10% of 124 (33)	91 ± 14
	6.5% of 538 (15)	97 [95–102]
	NR of 50 (16)	79 ± 17
Depression	38.1% of 126 (42)	39 ± 7
	31% of 402 (57) *ZSDS	31 ± 16
	30% of 179 (58)	61 ± 30
	29% of 287 (54)	Unclear
	19% of 58 (66)	70 [63–77]
	15% of 384 (56)	54 [47–59]
	12% of 124 (33)	91 ± 14
	11% of 402 (57) *BDI	31 ± 16
	11% of 105 (22)	46 [43–48]
	4.3% of 538 (15)	97 [95–102]
	NR of 50 (16)	79 ± 17
Anxiety or depression	46% of 91 (25)	183
	23% of 50 (18)	>28
	3% of 50 (17)	186 [175–199]
	NR of 50 (16)	79 ± 17
Dementia/memory loss	37.5% of 24 [(32) – ward]	>100
	29% of 287 (54)	Unclear
	20.8% of 96 [(32) – ICU]	>100
	20% of 50 (18)	>28
	15% of 227 (59)	Range 70–98
OCD	21% of 402 (57) *state anxiety	31 ± 16
	5% of 287 (54)	Unclear
Panic attacks	12% of 1,021 (18)	>28
Psychiatric morbidity	39% of 179 (58)	61 ± 30
Emotional symptoms	19.8% of 126 (42)	39 ± 7
	11.1% of 18 (30) *severe mood swings	85 (range 20–105)
	0.6% of 538 (15) *feelings of inferiority	97 [95–102]
Low QoL	72% of 124 (33)	91 ± 14
	67% of 227 (59) *QoL reduction	Range 70–98
	67% of 91 (25)	183
	59% of 210 (21) *CCQ	79 ± 17
	39% of 179 (58)	61 ± 30
	39% of 210 (21) *EQ5D,	79 ± 17
Dysphoria	1.7% of 538 (15)	97 [95–102]
Anorexia	NR of 180 (63)	125 (range 45–153)
**Cognitive**		
Loss of attention	50% of 18 (30)	85 (range 20–105)
	29.2% of 24 [(32) – ward]	>100
	24% of 1,021 (18)	>28
	16.7% of 96 [(32) – ICU]	>100
Confusion	21% of 183 (20)	35 ± 5
	NR of 451 (31)	117 (range 41–193)
Neurocognitive impairment	77% of 179 (58)	61 ± 30
	40% of 58 (66) *MoCA visuospatial	70 [63–77]
	40% of 105 (22) *impaired on MMSE	46 [43–48]
	28% of 58 (66) *MoCA total	70 [63–77]
	17.1% of 105 (22) *cognitive deficiency	46 [43–48]
	15% of 124 (33) *cognitive impairment	91 ± 14
	7% of 124 (33) *issues with cognitive function	91 ± 14
Concentration deficits	44.4% of 18 (30)	85 (range 20–105)
Short-term memory deficits	44.4% of 18 (30)	85 (range 20–105)
Word finding difficulty	27.8% of 18 (30)	85 (range 20–105)
Incoherent thoughts	5.6% of 18 (30)	85 (range 20–105)
**Sensory**		
Hyposmia/Anosmia/Dysnosmia (smell dysnfunction)	100% of 23 (35)	>30
	64.6% of 354 [(19) – Non-hospitalised]	79 ± 17
	60% of 35 (52)	~61
	59.8% of 112 [(19) – Hospitalised]	79 ± 17
	56% of 109 (47)	>30
	51.4% of 751 (45)	47
	51.3% of 187 (27) *taste and smell	28
	41% of 1,021 (18)	>28
	37% of 183 (20)	35 ± 5
	27.8% of 150 (44) *taste and smell	30
	22.7% 130 (44) *taste and smell	60
	21% of 227 (59) *anosmia-dygeisua	Range 70–98
	19% of 145 (68)	100
	14.8% of 143 (24)	>100
	14.6% of 24 [(32) – ward]	100
	14.4% of 139 (64)	Range 30–35
	12% of 451 (31)	117 (range 41–193)
	11% of 91 (25)	183
	11% of 1,733 (17)	186 [175–199]
	8.4% of 96 [(32) – ICU]	>100
	5.7% of 105 (22)	46 [43–48]
	4.6% of 238 (29)	122
	4% of 246 (23)	68 ± 16
	26% of 111 (61) *hyposmic	63 ± 46
	1% of 111 (61) *anosmic	63 ± 46
	NR of 90 (65)	61 [IQR 7]
	NR of 180 (63)	125 (range 45–153)
Hypogeusia/Ageusia/Dysgeusia (taste dysfunction)	65.2% of 112 [(19) – Hospitalised]	79 ± 17
	63.2% of 354 [(19) – Non-hospitalised]	79 ± 17
	50% of 109 (47)^$^	>30
	46% of 1,021 (18)	>28
	44% of 183 (20)	35 ± 5
	16.7% of 96 [(32) – ICU]	>100
	13% of 111 (61)	63 ± 46
	11.5% (64)	Range 30–35
	10% of 451 (31)	117 (range 41–193)
	10% of 143 (24)	60 ± 13
	9.4% of 24 [(32) – ward]	>100
	7% of 1,733 (17)	186 [175–199]
	7% of 111 (61)	63 ± 46
	5.7 % of 105 (22)	46 [43–48]
	5% of 238 (29)	122
	1.1% of 55 (71)	Range 64–93
	NR of 180 (63)	125 (range 45–153)
Dizziness/impaired vision/vertigo	49.6% of 354 [(19) – Non-hospitalised]	79 ± 17
	41.1% of 112 [(19) – Hospitalised]	79 ± 17
	17% of 287 (54)	Unclear
	12% of 187 (27)	28
	6% of 1,733 (17)	186 [175–199]
	6% 10% of 143 (24)	60 ± 13
	5% of 227 (59)	Range 70–98
	2.6% of 538 (15)	97 [95–102]
	NR of 451 (31)	117 (range 41–193)
Loss of appetite	14% of 187 (24)	28
	8% of 1,733 (17)	186 [175–199]
	7.9% of 143 (24)	60 ± 13
	NR of 1,939 (16)	79 ± 17
Eye irritation	22.0% of 354 [(19) – Non-hospitalised]	79 ± 17
	17.9% of 112 [(19) – Hospitalised]	79 ± 17
	20% of 183 (20)	35 ± 5
	10% of 143 (24)	60 ± 13
	4% of 1,021 (18) *conjunctivitis	>28
	NR of 451 (31) *conjunctivitis	117 (range 41–193)
Tinnitus	17% of 287 (54)	Unclear
	13% of 121 (60)	
Phonophobia	5.6% of 18 (30)	85 (range 20–105)
Chemosensory Dysfunction	39% of 46 (70)	Unclear
**Dermatological**		
Rash	3% of 1,733 (17)	186 [175–199]
	NR of 451 (31)	117 (range 41–193)
	NR of 180 (63)	125 (range 45–153)
Cutaneous signs	15.4% of 150 (44)	30
	11.5% of 130 (44)	60
	8% of 227 (59)	Range 70–98
Hair loss	28.6% of 538 (15)	97 [95–102]
	25% of 96 [(32) – ICU]	>100
	22% of 1,733 (17)	186 [175–199]
	18.8% of 24 [(32) – ward]	>100
**Functional**		
Mobility problems	56% of 91 (25)	183
	53.8% of 238 (29)	122
	40.3% of 201 (28)	91
	18% of 1,021 (18)	>28
	7% of 1,733 (17)	186 [175–199]
	NR of 1,939 (16)	79 ± 17
Personal care problems	13% of 91 (25)	183
	1% of 1,733 (17)	186 [175–199]
	NR of 1,939 (16)	79 ± 17
Usual activity problems	67% of 210 (21)	79 ± 17
	37% of 91 (25)	183
	2% of 1,733 (17)	186 [175–199]
	NR of 1,939 (16)	79 ± 17
Low 6MWT	23% of 1,733 (17)	186 [175–199]
	22.9% of 41 (72)	31 ± 9
	22% of 124 (33)	91 ± 14
	NR of 58 (66)	70 [63–77]
Low 2MWT	31.5% of 238 (29)	122
SPBB	22.3% of 238 (29)	122
Decreased functional status	64% of 124 (33)	91 ± 14
	62.6% of 91 (25)	183
	47.5% of 183 (69)	183
**Other**		
Renal failure	3% of 58 (66) *renal impairment	70 [63–77]
	1% of 287 (54)	Unclear
Constipation	NR of 1,939 (16)	79 ± 17
Sleep difficulties/Insomnia	61% of 384 (56)	54 [47–59]
	40% of 402 (57) *state anxiety	31 ± 16
	33.3% of 96 [(32) – ICU]	>100
	32% of 1,021 (18)	>28
	30.8% of 91 (25)	183
	30.2% of 24 [(32) ward]	>100
	28% of 145 (68)	100
	26% of 1,733 (17)	186 [175–199]
	17.7% of 538 (15) *sominpathy	97 [95–102]
	NR of 1,939 (16)	79 ± 17
Post-COVID Syndrome	41% of 227 (59)	Range 70–98
Liver Injury	11% of 58 (66) *blood tests,	70 [63–77]
	10% of 58% (66) *MRI	70 [63–77]
Sicca syndrome	12.8% of 143 (24)	60 ± 13
Flu-like symptoms	36.0% 130 (44)	30
	21.5% 150 (44)	60
Weight loss	37.5% of 112 [(19) – Hospitalised]	79 ± 17
	23.5% of 354 [(19) – Non-hospitalised]	79 ± 17
	17.2% 130 (44)	30
	15.9% 150 (44)	60
Red spots on feet	8% of 112 [(19) – Hospitalised]	79 ± 17
	4.3% of 354 [(19) – Non-hospitalised]	79 ± 17
Other	25.2% of 354 [(19) – Non-hospitalised]	79 ± 17
	17% of 112 [(19) – Hospitalised]	79 ± 17
Night Sweats	24% of 145 (68)	100
Seizure/cramps	NR of 451 (31)	117 (range 41–193)
Enlarged lymph nodes	NR of 451 (31)	117 (range 41–193)
Low fat free mass	19% of 124 (33)	91 ± 14
Sweating	23.6% of 538 (15)	97 [95–102]

*Prevalence data are representative of the follow-up time point (i.e., persistent symptoms) rather than initial classification at diagnosis of COVID-19*.

*** *, Table and text do not match; NR, reported as present in the cohort but no clear prevalence data*;

$ *, prevalence in people with dysgeusia as initial testing; BDI, Beck's depression inventory; CMR, cardiovascular magnetic resonance; EQ5D, EuroQol-5D: an instrument for measuring quality of life; IES-R, impact of events scale-revised; ICU, intensive care unit; CCQ, clinical chronic obstructive pulmonary disease (COPD) questionnaire; FVC, forced vital capacity; MMSE, mini-mental state exam; MoCA, Montreal cognitive assessment; MRI, magnetic resonance imaging; MWT, minute walk test; OCD, obsessive compulsive disorder; QoL, quality of life; SPPB, short physical performance battery; PTSD, post-traumatic stress disorder; ZSDS, Zung self-rating depression scale. Days are presented as mean ± standard deviation or median [interquartile range] or mean (range) if present in the original study*.

### Symptom Reporting

In total, 108 distinct symptoms were described by authors of the original articles, despite us grouping taste dysfunction, smell dysnfunction, and breathing problems together into three categories. There were 10 studies which reported cardiovascular symptoms, four which examined pulmonary symptoms, 25 which reported respiratory symptoms, 24 which reported pain-related symptoms, 21 which reported on fatigue of some description, 16 which reported general infection symptoms, 10 which reported symptoms of psychological disorders, 9 which reported cognitive impairment, 31 which reported a sensory impairment, seven which reported a dermatological complaint, 11 which reported a functional impairment, and 18 which reported a symptom which did not fit into any of the above categories.

Dyspnoea/breathlessness/shortness of breath/breathing problems (all one category) was the most reported symptom (27 cohorts), with smell dysfunction (26 cohort) second, fatigue/tiredness second (24 cohorts) third. Symptom prevalence varied significantly between studies, often from <10 to >70% (e.g., dyspnoea/breathlessness/shortness of breath/breathing problems, cough, sore throat, chest pain, headache, joint pain/arthralgia, pain or discomfort, fatigue, fever, neurocognitive impairment, smell dysfunction, and taste dysfunction).

### Study Location

Of the 50 studies, 37 were from Europe, four from North America, six from Asia, one from South America, one from Africa, and one where the location was unclear. Of the 37 studies from Europe, ten were conducted in Italy, five in France, five in Spain, five in the UK, two in the Netherlands, two in the Netherlands and Belgium, two in Germany, two in Greece, one in Austria, one in Denmark, one in Norway, and one in Ireland.

### Study Setting

Of the 50 studies, 27 concerned hospitalised individuals only, 13 were in both hospitalised and non-hospitalised combined, and three were in only non-hospitalised participants. The remaining studies were unclear as to whether participants were included or excluded based on whether they were hospitalised. Of the 27 studies concerning exclusively hospitalised participants, five exclusively studied participants from the ICU only, three were conducted in participants from the general ward only, and 19 that were explicitly in both ICU or general ward patients or were hospitalised but unclear whether to the ICU or general ward. For clarity, two studies had two cohorts (19, 32), and have been considered as individual data sets.

## Discussion

This scoping review examined the range of outcomes from studies pertaining to long COVID symptoms, aligned to our primary aim. Firstly, >100 symptoms have been reported by original investigations, which emphasises the diverse nature of long COVID. Secondly, the volume of articles published from 2020 onwards speaks to this rapidly emerging area of research. This review catalogues existing symptom literature, with a view to aiding physicians and healthcare practitioners better understand the range and prevalence of symptoms of long COVID. Moreover, we believe this information can facilitate discussion of research opportunities and issues that need to be addressed in future studies.

### Long COVID Symptoms and Their Prevalence

Results of this review support recent observations that long COVID can result in a wide variety of symptoms. From the studies included in this review, we identified more than 100 symptoms. A recent report by Davis et al. (73) similarly detailed over 200 symptoms in an international cohort of long COVID patients. The difference between their data and ours being largely explained by our grouping of similar classifications of symptoms (e.g., we grouped dyspnoea, shortness of breath, breathlessness as a single category). Nevertheless, this work supports the growing view that long COVID is typified by a disparate array of symptoms, across multiple physiological systems, and may often result in individuals experiencing their own idiosyncratic manifestation of the condition.

Unsurprisingly symptoms associated with acute COVID-19 infection appear most frequently in the literature, include sensory alterations, respiratory symptoms, chest pain, headaches, and fever. However, because of their association with acute infection (72, 74), it is difficult to determine the degree to which they occur in long COVID. Indeed, it is reasonable to assume that most studies designed their surveys to reflect acute symptoms. Thus, even though these categories are most commonly associated with long COVID, this may be due, in part at least, to them being the symptoms about which researchers most frequently enquired. Conversely, although not as commonly reported as acute symptoms, this review identified other common symptoms of long COVID, which are less closely aligned to acute COVID-19 infection. These include cognitive impairments, fatigue, neuralgia and myalgic pain, sleep difficulties, mobility impairments, and psychological symptoms (e.g., anxiety and depression). These findings support previous research reports (11, 56, 75), and case studies (76, 77) from which the defining characteristics of long COVID have emerged. It also supports prior work suggesting long COVID is a distinct condition rather than slowly resolving acute COVID-19 and associated symptoms (78).

### Heterogeneity in Prevalence

It was noted prevalence of symptoms displayed considerable divergence between investigations. A plausible *a priori* hypothesis would have been that heterogeneity in symptoms may be due to differences in study protocols or data collection methods (e.g., such as whether inclusion criteria required a prior confirmed COVID-19 test). However, there was limited evidence to support this view. For a variety of symptoms including dyspnoea, cough, sore throat, chest pain, headache, fatigue, and diarrhoea there was no clear pattern that explained observed heterogeneity. Studies reporting a high prevalence included online surveys of individuals self-reporting as having persistent symptoms (19), studies using in person evaluation of only those with a positive COVID-19 test (28) and studies using online surveys of both suspected and confirmed COVID-19 cases (21). Similarly, those reporting low prevalence also included self-reported COVID-19 infection (44) and those with positive PCR tests. Neither were there clear differences in duration of follow-up with similar follow up durations utilised in investigations reporting high [e.g., 74 days (21) −4 months (28)] and low [e.g., 60 days (44) −6 months (31)] prevalence. Taken together, these data suggests that, in these symptoms at least, long COVID is an inherently variable condition. While some symptoms are commonly considered to be associated with the condition (such as fatigue), they are by no means ubiquitous among patients. Practitioners should be aware of the idiosyncratic symptoms and experiences of people with the condition, which in turn will likely require personalised rehabilitation strategies. As an exemplar to emphasise this point, diarrhoea prevalence is a prime example of homogeneity in study characteristics yet heterogeneity in results. The greatest prevalence was reported by Dennis et al. [(28); 59%], and the lowest by Bellan et al. [(29); 1%]. These studies have similar samples sizes (*n* = 201 vs. *n* = 238), similar follow-up durations (3 vs. 4 months), similar study design (cohort), both studies considered only confirmed cases of COVID-19, and both studies considered hospitalised participants [Bellan et al (29) considered exclusively hospitalised patients whereas Dennis et al. (28) considered both hospitalised and non-hospitalised] individuals. Both studies were robust in research design, with few difference in methodology, yet divergence in prevalence of diarrhoea was reported.

In addition, it is also worth noting there was no discernible pattern concerning participants who had a confirmed COVID-19 infection vs. those with suspected COVID-19 (but who may not have been tested at the time of infection). This finding is supported by studies which have specifically investigated confirmed vs. suspected cases [e.g., Meys et al. (21)]. Consequently, it may not be necessary in future studies to have a positive COVID-19 test as an inclusion criterion, since the symptom range (and variation) appears to be similar in both confirmed and suspected cases. This is particularly useful finding for researchers and patient groups given that, particularly early in the pandemic, testing was unlikely to have taken place, despite obvious acute symptoms. A caveat to this suggestion is that although this was applicable to those infected with COVID-19 in 2020/2021, this may not be true for 2021/2022 when other viruses (e.g., influenza) may be circulating to a greater extent in the population the addition of a positive test as an inclusion criteiron may be necessary to exclude other potential causes of post-viral symptoms.

Whilst considerable variance between studies was evident, variation *within* each study in terms of its prevalence rank in Table 2 was small. By this, we mean prevalence rates may be related to some unknown, cohort-specific factor as whole study cohorts were relatively consistent when studies were ranked by their reported symptom prevalence. For example, for the 15 symptoms they have reported, Dennis et al. (28) had the greatest prevalence in eight categories and were in the top three for the remainder. Similarly, Goertz et al. (19) reported on two cohorts (hospitalised and non-hospitalised), and frequently report some of the highest incidence rates for the symptoms they assessed. Conversely, Bellan et al. (29) reported 14 symptoms and for 10 of those they consistently report one of, or the, lowest prevalence rates, (and for two of the remainder they are the only reporting study, so comparisons are unfeasible). It is difficult to speculate from the available data what specific factors are explanatory in this context. Some potential factors include differences in geographic location, treatment algorithms, cohort profile (e.g., existing co-morbidities). However, further longitudinal studies will be required to provide a more comprehensive assessment of risk factors for long COVID.

### Study Characteristics and Methodologies

In relation to our second objective, studies included were mostly cohort studies or cross sectional studies, which are both observational studies (79). We chose to report study design as reported by the authors of the original investigation but often these studies utilised the same research design in that several individuals who had recovered from acute COVID-19 were contacted and asked for their symptom at that time point. Thus, we would suggest that in many cases, authors who defined their study as ‘cross sectional' had actually conducted retrospective cohort studies (79).

Follow-up periods ranged from 4 weeks to approximately 6 months. As mentioned previously, it would have been reasonable to speculate *a priori* that this influenced symptom prevalence as some symptoms may have been evident at 4 weeks but resolved by 6 months. However, follow-up period had little effect on prevalence differences *between* studies. Conversely, within each cohort, following participants for a greater time course may have influence *within* study prevalence rates. This is an inherent problem with a single follow-up point, which several of the cohort studies in this review utilised. It is possible that some research groups involved in the articles included in this review will have research projects ongoing, continually detailing symptoms which would permit dissemination of information concerning time course of symptom resolution.

Studies were conducted worldwide (Europe; *n* = 37, North America; *n* = 4, Asia; *n* = 6, South America; *n* = 1, Africa; *n* = 1, location unclear; *n* = 1). Whilst the geographical location of investigations conducted may be of surprise to some because COVID-19 originated in China and therefore the healthcare system of China had greatest potential for follow-up duration, Europe has to date experienced the most absolute number of confirmed cases. This was likely the fact the Chinese government implemented more drastic lockdown measures than did European governments. This likely attenuated virus transmission and is evidenced by China having ~95,000 confirmed cases at the time of writing, whilst the UK has had >7.3 million confirmed cases.

Sample sizes ranged from 12 to 1,939, with 16 studies having n <100. This supports our rationale to scope the literate rather than to meta-analyse the field, as we feel reporting prevalence as a percentage of 19 individuals is not epidemiologically valid [e.g., Woo et al. (30)]. As mentioned previously, it would have been reasonable to speculate *a priori* that this influenced symptom prevalence but as mentioned above, this was not the case.

### Recommendations for the Advancement of the Investigative Area

In relation to our third objective, we believe the investigative area concerning long COVID could be improved by greater methodological detail. As evidenced from Table 1, we were often unable to extract details concerning methods utilised which may have influenced results, and thus interpretations. For example, given the known effect of chronological age on acute COVID-19 severity (1), we believe this information should be present in methods of articles included in this review, although this was not always the case. On the topic of age, there is now some emerging evidence that children may experience similar long-term effects to adults after COVID-19 infection (80–83). Whilst we did not specifically exclude studies on the basis of age, it is evident from Table 1 that few studies were conducted in children. Thus, long COVID in children may be an area for further exploration.

To improve the investigative area in the future, serial longitudinal follow-ups within each cohort would allow for information around time course of symptoms. We believe this would assist physicians better understand the prevalence of symptoms at each relevant time point (e.g., whether sensory dysregulation is typically present at 1 month, 2 months, or 3 months post-acute COVID-19 recovery). However, this may be labour-intensive so remote symptom tracking using mobile technology may prove advantageous in this context. This would alleviate resource commitments associated with data collection but may result in greater time and expense concerning data management and analysis. Finally, and most importantly, precision of reporting follow-up timing, prevalence of comorbidities, and setting (i.e., outpatients' clinic, smell and taste clinic) would all enhance the existing literature base.

## Conclusions

In conclusion, this review catalogued the range and prevalence of symptoms of long COVID. We report the most reported symptoms fell into categories of sensory, respiratory, pain, and fatigue respectively. Prevalence of each symptom varied significantly, but unlikely because of study heterogeneity, and appeared to be related to unknown cohort-specific factors. By this, we mean that study design, participant age, study setting, participant sex, and follow-up duration did not appear to explain differences in symptom prevalence, but instead prevalence differed from one study to another, despite methodological similarities in some instances. It is expected that as the investigative area advances and more is known about the long COVID condition, a regression towards the mean will occur and a better knowledge of symptom prevalence will arise.

## Data Availability Statement

The raw data supporting the conclusions of this article will be made available by the authors, without undue reservation.

## Author Contributions

LH, JI, and NS: conceptualisation, methodology, formal analysis and investigation, writing—original draft preparation, writing—review and editing, visualisation, project administration, and funding acquisition. All authors contributed to the article and approved the submitted version.

## Funding

This work was supported by a grant from the Chief Scientist Office (grant no COV/LTE/20/08).

## Conflict of Interest

The authors declare that the research was conducted in the absence of any commercial or financial relationships that could be construed as a potential conflict of interest.

## Publisher's Note

All claims expressed in this article are solely those of the authors and do not necessarily represent those of their affiliated organizations, or those of the publisher, the editors and the reviewers. Any product that may be evaluated in this article, or claim that may be made by its manufacturer, is not guaranteed or endorsed by the publisher.
